# Bacteremia affects the mortality of septic patients with high serum procalcitonin level in the ICU

**DOI:** 10.1186/cc10668

**Published:** 2012-03-20

**Authors:** M Kamochi, K Nagata, Y Isa, S Nihei, N Harayama, K Aibara, T Sata

**Affiliations:** 1 University Hospital of Occupational and Environmental Health, Kitakyushu, Japan

## Introduction

It is still controversial whether bacteremia affects the severity and the mortality of septic shock. Recent diagnostic criteria of septic shock do not include the presence of bacteremia, because rapid diagnosis and immediate treatment are necessary to improve the survival of septic patients. However, the presence of bacteremia seems to relate to the severity and mortality of septic shock patients in the ICU.

## Methods

The patients clinically suspected with sepsis were tested for serum procalcitonin level using a procalcitonin kit (BRAHMUS PCT Kit). The PCT test was performed 334 times from March 2008 to August 2010. Sixty-three adult patients showed high PCT level (>10 ng/ml). Thirty of 62 (48%) patients showed bacteremia. Sixteen of these bacteremic patients were Gram-negative bacteremia and 14 patients were Gram-positive bacteremia. The hemodynamic parameter, APACHE II score, SOFA score, serum lactate, some other laboratory data and mortality rate were compared between the patients with bacteremia and those without bacteremia. Statistical analyses were performed by chi-square test and Mann-Whitney U test.

## Results

The bacteremic patients with high serum PCT level showed significant higher APACHE II score, SOFA score and serum lactate concentration than nonbacteremic patients. The mortality rate of bacteremic patients was significantly higher than that of nonbacteremic patients (66% vs. 28.1%, *P *< 0.01). There were no differences in the severity and the mortality between Gram-negative and Gram-positive bacteremia.

## Conclusion

The presence of bacteremia relates to the severity and the mortality of septic patients with high serum PCT in the ICU.

